# Quercetin prevents spinal motor neuron degeneration induced by chronic excitotoxic stimulus by a sirtuin 1-dependent mechanism

**DOI:** 10.1186/s40035-017-0102-8

**Published:** 2017-11-21

**Authors:** Rafael Lazo-Gomez, Ricardo Tapia

**Affiliations:** División de Neurociencias, Instituto de Fisiología Celular, Universidad Nacional Autónoma de México, Circuito exterior s/n, Ciudad Universitaria, Coyoacán, 04510 Ciudad de México, Mexico

**Keywords:** Motor neuron disorder, Quercetin, Resveratrol, Sirtuin, Excitotoxicity

## Abstract

**Background:**

Excitotoxicity is a mechanism of foremost importance in the selective motor neuron degeneration characteristic of motor neuron disorders. Effective therapeutic strategies are an unmet need for these disorders. Polyphenols, such as quercetin and resveratrol, are plant-derived compounds that activate sirtuins (SIRTs) and have shown promising results in some models of neuronal death, although their effects have been scarcely tested in models of motor neuron degeneration.

**Methods:**

In this work we investigated the effects of quercetin and resveratrol in an in vivo model of excitotoxic motor neuron death induced by the chronic infusion of α-amino-3-hydroxy-5-methyl-4-isoxazolepropionic acid (AMPA) into the rat spinal cord tissue. Quercetin and resveratrol were co-infused with AMPA and motor behavior and muscle strength were assessed daily for up to ten days. Then, animals were fixed and lumbar spinal cord tissue was analyzed by histological and immunocytological procedures.

**Results:**

We found that the chronic infusion of AMPA [1 mM] caused a progressive motor neuron degeneration, accompanied by astrogliosis and microgliosis, and motor deficits and paralysis of the rear limbs. Quercetin infusion ameliorated AMPA-induced paralysis, rescued motor neurons, and prevented both astrogliosis and microgliosis, and these protective effects were prevented by EX527, a very selective SIRT1 inhibitor. In contrast, neither resveratrol nor EX527 alone improved motor behavior deficits or reduced motor neuron degeneration, albeit both reduced gliosis.

**Conclusions:**

These results suggest that quercetin exerts its beneficial effects through a SIRT1-mediated mechanism, and thus SIRT1 plays an important role in excitotoxic neurodegeneration and therefore its pharmacological modulation might provide opportunities for therapy in motor neuron disorders.

**Electronic supplementary material:**

The online version of this article (10.1186/s40035-017-0102-8) contains supplementary material, which is available to authorized users.

## Background

Motor neuron disorders (MNDs) are a heterogeneous group of neurodegenerative diseases pathologically characterized by the loss of MNs, which notably include amyotrophic lateral sclerosis (ALS) and spinal muscular atrophy (SMA). Chronic MN loss leads to gradually increasing weakness and paralysis, and ultimately to respiratory failure and death. These disorders are uniformly fatal, thus it is a priority to characterize the basic mechanisms that cause MN degeneration as a mean to develop appropriate therapeutic interventions [1].

Glutamatergic excitatory neurotransmission is a fundamental process in the mammalian brain, but an excessive activation of glutamate receptors results in excitotoxic neuronal death [2]. Although several hypotheses have been advanced to explain the selective degeneration of MNs, there is ample evidence of a role of excitotoxicity in MND, especially for ALS, although a causal relationship remains to be established (for in-depth reviews see [3, 4]). For example, increased concentrations of glutamate have been found in cerebrospinal fluid of ALS patients [5], and in synaptosomes obtained from human mutated superoxide dismutase G93A gene (hSOD1^G93A^) transgenic mice, an animal model of familial ALS, an increased basal release of glutamate has been documented [6, 7].

Our group designed an in vivo experimental model of chronic MN death in healthy rats through the infusion of α-amino-3-hydroxy-5-methyl-4-isoxazolepropionic acid (AMPA) directly in the lumbar spinal cord, using osmotic minipumps. With this model we replicated several important characteristics of MND: its chronic course, its onset in adulthood, and the fact that most of the cases are sporadic [8]. This approach has allowed us to study some of the basic mechanisms underlying excitotoxic MN death [9–11], and also to test potential strategies for neuroprotection, such as growth factors [12, 13] and energy substrates [14, 15].

Polyphenols are plant-derived compounds that have emerged as a promising therapeutic approach for several neurodegenerative disorders [16, 17]. This beneficial effect has been attributed to the allosteric activation of sirtuin 1 (SIRT1), the first member of the class III of oxidized nicotine adenine dinucleotide (NAD^+^)-dependent histone deacetylases [18].

Among the vast amount of polyphenolic compounds discovered, resveratrol (RSV) and quercetin (QCT) are two of the most promising molecules. RSV is a well-documented sirtuin activating compound (STAC), and has shown neuroprotective effects in in vitro and in vivo models of excitotoxic neuronal degeneration [19]. QCT has been shown to reduce glutamate-induced hippocampal [20] and retinal [21] neuronal death in vitro, as well as neuronal damage in an in vivo model of focal cerebral ischemia [22]. In addition, it was recently reported to be protective in a model of hypoxia-mediated hippocampal neurodegeneration by modulating SIRT1 expression [23]. However, studies on the effects of these compounds in experimental models of MND are scarce. Therefore, we explored the possible neuroprotective effects of RSV and QCT in our established model of chronic AMPA-induced excitotoxic spinal MN degeneration in vivo, and the involvement of SIRT1 as the mechanism of neuroprotection exerted by these polyphenols.

## Methods

### Animals

Adult Wistar male rats (280–300 g) were used in all of the experiments and were handled in accordance with the Rules for Research and Health Matters (Mexico), with international standards of research animal welfare (including ARRIVE guidelines), and with approval of the Institutional Committee for the Care and Use of Laboratory Animals (protocol approval number RTI21–14). All animals were housed in a controlled laboratory environment with a 12 h light/dark cycle, and fed with regular animal chow and water ad libitum. All surgical procedures were performed under general anesthesia, and every effort was made to minimize the number of animals used, as well as animal suffering during experimental procedures.

### Drugs and osmotic minipump preparation

All drugs were dissolved in vehicle solution that consisted in a mixture of isotonic saline solution and 3% *v*/v dimethylsulfoxide. Osmotic minipumps (Alzet model 2004, volume ~250 μL, flow rate 6 μl/day; Durect, Cupertino, CA, USA) were filled with the indicated solutions, which were vehicle only (control group), AMPA [1 mM], and/or RSV, QCT and EX527, at concentrations calculated in such a way that the osmotic minipump delivered 1 nanomole/day (nm/d) or 10 nm/d, or a mixture of drugs, as indicated in Results.

Our group has previously reported that chronic infusion of AMPA into the rat spinal cord in vivo induces paralysis, MN degeneration and astrogliosis [12, 24]. In these studies a 7.5 mM concentration was used and this caused a relatively rapid (~3 days) paralysis and MN death, which makes testing potential neuroprotective drugs difficult. Therefore, we selected a lower concentration (1 mM) that we had previously shown to produce a more gradual and reproducible paralysis, MN degeneration and spinal cord gliosis, features characteristic of MNDs [12]. The concentrations of the remaining drugs were chosen on the basis of preliminary experiments. (R,S)-AMPA was purchased from Tocris Bioscience (Bristol, United Kingdom), and RSV, QCT and EX527 from Sigma Aldrich (St. Louis, MO, USA).

### Surgical procedures for osmotic minipump implantation

Osmotic minipumps were prepared 48 h before surgical implantation, and were incubated at 37.0 ° C in saline solution for flow rate stabilization. The procedure for osmotic minipump implantation was performed essentially as previously described [12], with minor modifications. Briefly, animals were anesthetized with 5.0% isofluorane in a 95% O2/5% CO2 mixture and placed in a spinal unit, and isofluorane concentration was gradually diminished to 1.5–2.0% as the surgery was performed. After shaving and disinfection, a median sagittal incision, ~4 cm long, was made in the back and the underlying fascia and muscle tissue were dissected until appropriate visualization of the L3 vertebral lamina was achieved. The spinous process was removed with a drill, and a ~2 mm diameter hole was drilled in the right lamina until the spinal cord tissue was visualized and the meninges were carefully removed with a metallic hook. A stainless-steel screw (3.7 mm long, 1 mm diameter) was fixed in the left lamina. A fused silica capillary (1 mm long, 50 μm internal diameter, 80 μm external diameter; VitroCom Inc., Mountain Lakes, NJ, USA) was carefully advanced down into the spinal cord in a vertical fashion 0.8 to 1 mm; this capillary was attached to a plastic tube (cannula) that was connected, and fixed with cyanoacrylate glue, to the osmotic minipump. During all these procedures great care was taken to avoid unnecessary damage to the spinal cord tissue. Dental cement was poured and let dry on the L3 vertebra, to fix both the screw and the cannula on place. Osmotic minipumps were subcutaneously placed in the back of the animal, at the right side of the vertebral column. Finally, the skin incision was closed with surgical stainless-steel clips, anesthesia was withdrawn, and animals received a single intraperitoneal antibiotic shot and were monitored until recovery (see Additional file 1: Figure S1A for details). In addition to control group, where vehicle was infused into the spinal cord tissue, a group of sham surgeries was performed to assess the potential effect of the cannula’s glass capillary insertion into the spinal cord on the proliferation of astroglial and microglial cells. These surgical procedures were identical to those previously described until laminectomy completion, except no cannula and no osmotic minipump were implanted. Animals subjected to these sham surgeries were perfused/fixed, as described below, 10 days after the procedure.

### Behavioral assessment

Four to five days prior to surgery, rats were trained to walk during 120 s on an accelerating Rotarod (Columbus Instruments, Columbus, OH, USA), starting from 10 rpm (0.2 rpm/s of acceleration). Time to fall from the instrument was recorded, up to a limit of 120 s. Also, grip strength of both hind limbs was measured by placing the animals on their hind limbs on the metallic mesh of a grip strength meter (TSE Systems, Chesterfield, MO, USA) and gently pulling the tail to induce the animals to escape from the examiner; data was disregarded if the animals, when attempting to escape, used their forelimbs. The maximum force displayed by the instrument in every trial was recorded in ponds, and normalized to values obtained the day before surgery (day 0). In both tests the best time, or the greater force, out of three trials was recorded. Great care was taken to avoid excessive distress of the animals, and appropriate time between trials was given to animals for rest. These behavioral motor tasks were assessed daily for 10 days and, on this cutoff time, animals were sacrificed and fixed/perfused for histology.

### Perfusion/fixation and Nissl staining

Behavioral assessment was concluded 10 days after minipump implantation, and rats were perfused and fixed for histological analyses as previously described [12]. Briefly, animals were deeply anesthetized with an intraperitoneal injection of pentobarbital, the rib cage was cut to expose the heart, and a wide cut in the right atria was made. A needle, connected to a peristalsis pump, was inserted into the left ventricle and ~250 ml of ice cold normal saline were perfused, followed by ~250 ml of 4% paraformaldehyde in 0.1 M phosphate buffer. The back was dissected, the acrylic implant removed, and the lumbar spinal cord tissue was obtained by pushing it out of the vertebral canal with cold saline solution in a syringe. Tissue was postfixed for 48 h at 4.0 ° C, then dehydrated in increasingly concentrated sucrose solutions (10, 20 and 30%), and the region where the cannula was inserted was visually identified and used for study. Approximately 100 transverse sections (40 μm thick) were obtained in a cryostat, and the slices that showed the cannula’s capillary glass track were used for analyses and, of these, 6–8 were processed for immunocytochemistry (see below), and 15 to 20 slices were stained with cresyl violet (Nissl staining) (for further details see Additional file 1: Figure S1B). The number of morphologically healthy MNs (multipolar neurons with clear cytoplasm, soma diameter > 20 μm and distinguishable nucleus) was counted in the ipsilateral and contralateral ventral horns (for a histological comparison of healthy and degenerating MNs see Additional file 1: Figure S1C). Micrographs were obtained with digital Nikon camera attached to a Nikon Eclipse optic microscope (Melville, NY, USA), with a 10X objective, of the ventral gray matter. All images were minimally manipulated *off-line*, with cropping and brightness/contrast adjusted to ensure better visualization of MNs.

### Immunocytochemistry and confocal microscopy

To study the expression pattern of SIRT1 in the spinal cord tissue, double immunocytochemistry was performed on floating tissue sections of intact rats for SIRT1 and microtubule associated protein 2 (MAP2), ionized calcium-binding adapter molecule 1 (Iba1), or glial fibrillary acidic protein (GFAP). To perform glial cell counting in treated animals, double immunocytochemistry was performed for GFAP and Iba1 on floating tissue sections. First, sections were permeabilized with PBS 0.1 M/Triton X-100 0.3% *v*/v solution for 10 min; all subsequent procedures were carried on this solution. Then, tissue sections were blocked with bovine albumin 5% *w*/*v* for 120 min, and later incubated in the same blocking solution with primary antibody at 4.0 °C for 48 h with gentle shaking. Primary antibodies were used at the following dilutions: chicken anti-MAP2, 1:1000; chicken anti-GFAP, 1:1000; rabbit anti-Iba1, 1:500; and mouse anti-SIRT1, 1:50. All primary antibodies were purchased from Abcam (Cambridge, MA, USA). Later, primary antibodies were washed thrice, and antibody binding was revealed with the following secondary antibodies in the indicated dilutions: goat anti-chicken IgY FITC, 1:200; donkey anti-rabbit IgG Alexa Fluor 647, 1:200; goat anti-mouse IgG FITC, 1:200. All secondary antibodies were purchased from Life Technologies (Waltham, MA, USA). Tissue sections were exposed for 120 min to secondary antibodies in the dark and at room temperature, and then they were washed thrice before mounting in xylene-treated glass slides with simple fluorescent mounting medium (Dako Inc., Carpinteria, CA, USA, for glial cell counting) or with DAPI-containing mounting medium (Vector Laboratories; Burlingame, CA, USA, for SIRT1 location assessment). Fluorescence imaging was performed in a Zeiss LSM 710 (Oberkochen, Germany) confocal microscope. Imaging parameters (laser intensity, gain, digital offset, confocal aperture) were manually adjusted initially on tissues obtained from control group, and later on used on all other preparations. For glial cell counting, stacks were composed of images obtained every 2.5 μm that spanned the complete thickness of the tissue, with a 20X objective, of the ventral gray matter, composed of two channels: green for GFAP/FITC imaging and red for Iba1/AlexaFluor 647 imaging. Maximal intensity projections and merged images were obtained off-line with FIJI program [25].

### Glial cell counting analysis off-line

An .lsm format composite image was obtained for each side of each slice of spinal cord tissue. At least 3 slices were analyzed per animal of 5 animals per group. Since we observed that cannula insertion, even in control groups, induced astrogliosis (although no microgliosis), we only used the data obtained from the contralateral side to cannulae insertion for analysis. To perform the automated counting of GFAP(+) and Iba1(+) particles, interpreted as astrocytes and microglial cells respectively, an Image J [26] programming language-based macro was designed. This macro instructed FIJI to open and split.lsm image channels (as described above, green for GFAP(+) particles and red for Iba1(+) particles), and to create and save composite 16-bit depth TIFF format images in separate folders, one for GFAP and one for Iba1. Then these TIFF images were subjected to Z-stacking, smoothing, and automated thresholding with the Max Entropy method. Binary versions of each Z-stack TIFF image were obtained, and automated particle analysis was carried on. For astroglial cells, GFAP(+) particles >10 μm^2^ were counted, and for microglial cells, Iba1(+) particles >19.5 μm^2^ were counted. This threshold was chosen based on the profile of the whole-particle frequency histograms displayed by the Analyze Particles tool of FIJI. In addition, a researcher, blinded to the results of this study, independently and manually counted a sample of the images, with comparable results obtained to those of the automated counting. Also, this macro obtained the area of tissue photographed, using the GFAP/FITC channel TIFF images previously obtained. These images were Z-stacked, smoothened, and the Moments method of automated thresholding was applied. Then, the whole area of the image was quantified with the Measure tool. The number of particles in an area of (μm^2^ × 10^5^) was used to calculate the mean values and standard deviation for each animal.

### Statistical analysis

All statistical analyses were carried out in GraphPad Prism 5.0 (La Jolla, CA, USA). After checking data (time to fall, normalized grip strength, MN counting, GFAP+ particle counting and Iba1+ particle counting) followed a normal distribution with the D’Agostino-Pearson omnibus normality test, parametric versions of hypothesis testing were performed. A two-way ANOVA (in the case of behavioral tasks) followed by Tukey’s post hoc test, or a one-way ANOVA (for number of healthy MN, and glial cell counting) followed by Tukey’s post hoc test were used. A value of *p* < 0.05 was considered statistically significant.

## Results

### SIRT1 is expressed in neuronal cells in the spinal cord, but is absent in glial cells

**Fig. 1:**
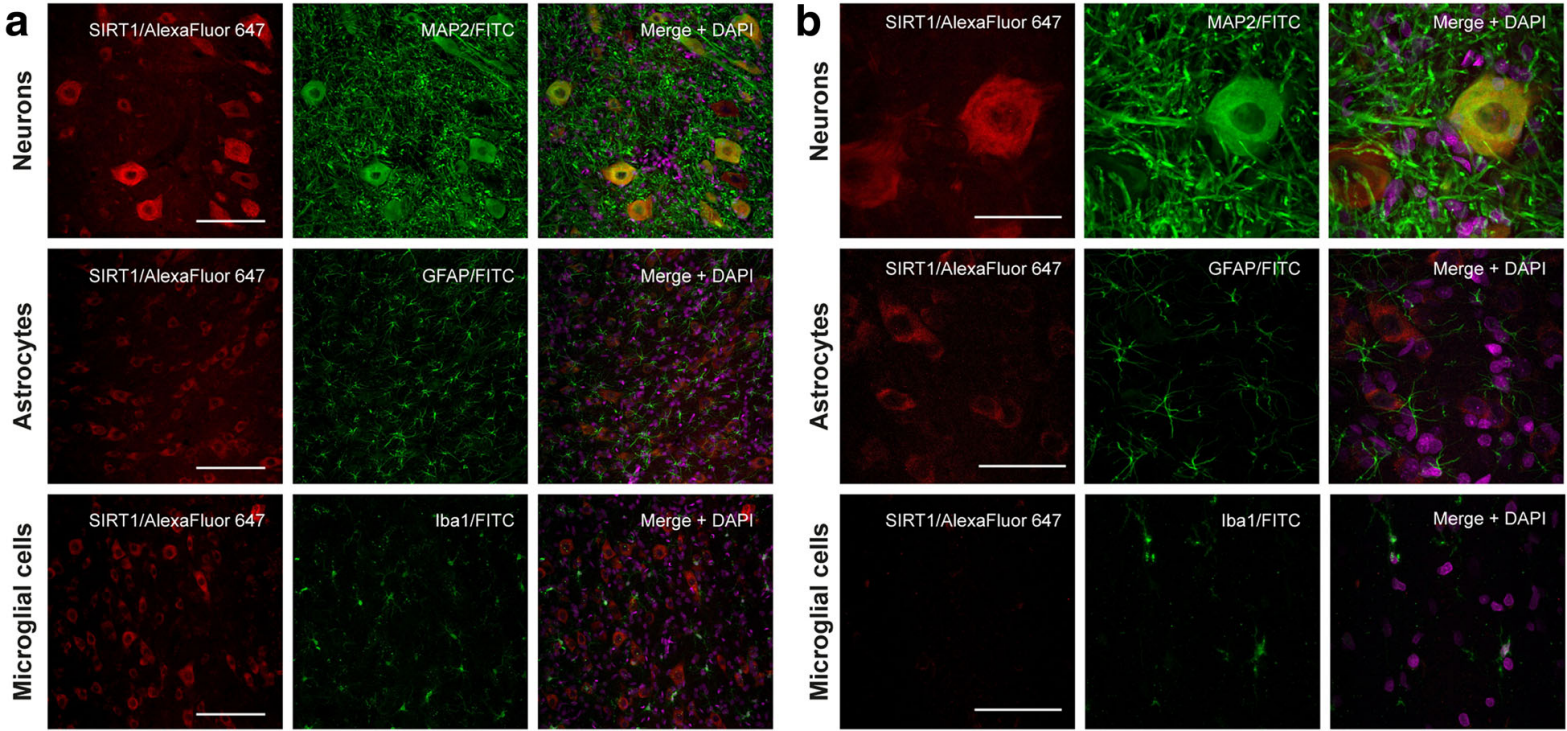
SIRT1 is expressed in lumbar spinal neuronal somas, including MNs. **a** Representative maximal intensity projections of confocal immunohistofluorescence images of the ventral gray matter of spinal cord sections of intact rats labeled with anti-SIRT1 (red, left and right columns), anti-MAP2 (green, Neurons row), anti-GFAP (green, Astrocytes row) and anti-Iba1 (green, Microglial cells row). Merged images (right column, with DAPI staining in magenta), show that there is co-location of SIRT1 signal only in neuronal somas and nuclei, including MNs (yellow cells). Scale bar, 100 μm. **b** Representative maximal intensity projections of high-magnification confocal immunohistofluorescence images; labeling and color codes are the same as in panel A. In the merged images SIRT1 labeling co-localizes with MAP2 immunostaining (Neurons rows) and is also present in neuronal nuclei. SIRT1 labeling signal was not detected in GFAP (Astrocytes row) or in Iba1 (Microglial cells) immunostained cells. Scale bar, 50 μm. Images were obtained from three spinal cord slices of five animals, all of which yielded similar results

We performed double immunohistofluorescence in the spinal cord tissue slices of intact rats to describe the cellular location of SIRT1 expression. We observed that SIRT1 is widely expressed in the ventral gray matter of the spinal cord, especially in the soma and nuclei (DAPI-labeling) of MAP2-positive cells (neurons), but it was not found in GFAP(+) (astrocytes) and Iba1(+) (microglia) cells. (Fig. 1).

### Motor behavior tasks changes induced by AMPA, RSV, QCT or EX527

**Fig. 2:**
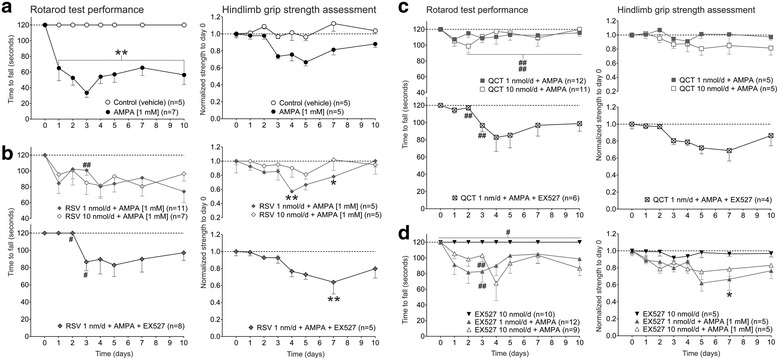
Effects of RSV, QCT and EX527 on motor behavior tasks during chronic AMPA-induced excitotoxicity. **a** Results of rotarod test and hindlimb grip strength measurement, as indicated, during 10 days of continuous vehicle (control) or AMPA infusions. **b** RSV effects, alone or co-infused with AMPA and EX527, as indicated. **c** QCT effects, alone or co-infused with AMPA or EX527. **d** Effects of EX527 alone or co-infused with AMPA. Number of animals is shown in parentheses. *p < 0.05 and **p < 0.01 versus control, and ^#^ *p* < 0.05 and ^##^ *p* < 0.01 versus AMPA. Two-way ANOVA followed by Tukey’s post hoc test

The results of treatment with 1 mM AMPA for 10 days replicated previously reported data [12]. Treated animals showed a gradual, but incomplete, paralysis of the hindlimbs, beginning in the ipsilateral limb and later involving the contralateral one. This change was different from control group performance from day 1 until day 10 of infusion (Fig. 2a, left panel). Also, it is noteworthy that time to fall abruptly diminishes during the first three days (from 65.0 ± 16.0 s at day 1 until 33.5 ± 5.9 s at day 3), and then reaches a plateau value at ~57 s until day 10. As opposed, in the hindlimb grip strength assessment AMPA treatment did not induce significant strength reductions, although a trend towards lower values was observed (~33% reduction from basal conditions at day 5; Fig. 2a, right panel).

RSV-alone infusion (at 10 nm/d) did not change motor behavior performance, as assessed by rotarod or hindlimb strength changes (data not shown). RSV, either at 1 nm/d or at 10 nm/d, co-infused with AMPA [1 mM] did not prevent the AMPA-induced decline in rotarod test performance, except for a significant protection (*p* < 0.01) at day 3 with the 1 mM concentration. We further explored the potential contribution of SIRT1 activation by RSV, so we used the very potent and selective inhibitor EX527 [27]. We co-infused RSV at 1 nm/d and AMPA [1 mM] with EX527 at 10 nm/d to ensure SIRT1 remained inhibited while RSV exerted its effects. This co-infusion resulted in a slight and transitory improvement in the time to fall from rotarod as compared to AMPA (*p* < 0.05 only at days 2 and 3), but was not different from the RSV 1 nm/day + AMPA group values (Fig. 2b, left panels). No changes in hindlimb grip strength were observed, although statistical significance was reached as compared to control at days 4 and 7 in the RSV 1 nm/d + AMPA group, and at day 7 in the RSV 1 nm/d + AMPA + EX527 group (Fig. 2b, right panels).

QCT-alone infusion did not induce reductions in time to fall or strength loss throughout the 10 days of infusion (data not shown). We also found that at both doses tested, 1 and 10 nm/d, QCT almost completely prevented the AMPA-induced paralysis, being significantly different from day 2 and throughout the 10 days of infusion (*p* < 0.01 versus AMPA for both concentrations), as assessed by rotarod. SIRT1 role in the QCT-mediated effects was also studied with EX527 at 10 nm/d. EX527 added to the infusion of QCT 1 nm/day + AMPA resulted in a reduced time to fall at days 2 and 3 when compared to AMPA (*p* < 0.05). However, when compared to QCT 1 nm/day + AMPA group values, a clear trend to diminished time to fall is observed from day 4 until day 10, although no signficance was reached (Fig. 2c, left panels). In the hindlimb grip strength assessment (Fig. 2c, right panels), QCT-infusion resulted in no significant differences respect to AMPA or to control values, and a clear trend to reduced strength is noted when EX527 is added to QCT 1 nm/day + AMPA treatment, as well.

EX527-alone treatment did not result in reductions in the time to fall in rotarod test or in the hindlimb strength assessment (Fig. 2d, left panel). EX527 co-infused, at 1 and 10 nm/d, with AMPA did not show an increase in the time to fall from rotarod as compared to AMPA value, although a significant increased value was observed at day 3 (*p* < 0.01). No significant differences were appreciated in the hindlimb grip strength assessment in any group studied, although a clear trend toward reduced strength is observed in both doses when compared to control values (*p* < 0.05 only at day 7) (Fig. 2d, right panels).

### Effects of RSV, QCT or EX527 on AMPA-induced MN degeneration

**Fig. 3:**
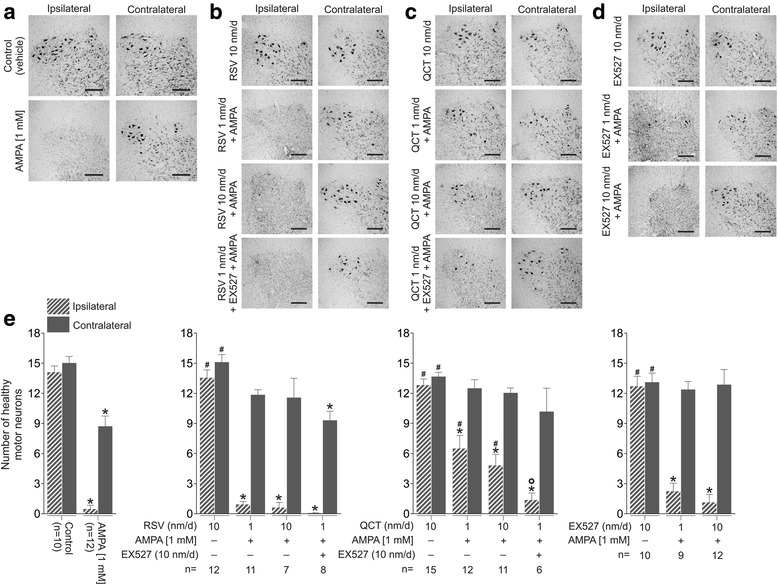
MN degeneration is prevented by QCT infusion, but not by RSV. Representative photomicrographs of Nissl stained ipsilateral and contralateral lumbar ventral spinal cord of rats after 10 days of continuous infusion of: **a** vehicle or AMPA; **b** RSV alone or co-infused with AMPA and EX527; **c** QCT alone or co-infused with AMPA and EX527; **d** EX527 alone or co-infused with AMPA. Scale bar, 250 μm. **e**. Quantitative analysis of healthy motor neurons. Number of animals is shown in parentheses. *p < 0.05 versus control, ^#^ *p* < 0.05 versus AMPA, and °*p* < 0.05 versus QCT 1 nm/d + AMPA. One-way ANOVA followed by Tukey’s post hoc test

An almost complete absence of MNs in the ipsilateral side to the infusion was observed in Nissl stained sections of the spinal cords of animals treated with AMPA after 10 days of continuous infusion, whereas in the contralateral side only a modest loss was evident (Fig. 3a). When quantified, more than 90% of MNs were lost in the ipsilateral side in the AMPA group (0.4 ± 0.3 versus 14.1 ± 0.6), whereas in the contralateral side remained approximately 60% of MNs as compared to control (8.7 ± 1.0 versus 15.0 ± 0.6, *p* < 0.01; Fig. 3e, left histogram).

RSV-alone infusion did not change ventral spinal cord histology or MN number. RSV did not prevent the neurodegeneration provoked by AMPA after 10 days of infusion (Fig. 3b). MN quantification revealed no changes, both ipsilateral (0.9 ± 0.3 for RSV 1 nm/d and 0.6 ± 0.5 for 10 nm/d) and contralateral (11.8 ± 0.5 for RSV 1 nm/d and 11.5 ± 1.9 for 10 nm/d) to the infusion, when compared to AMPA (Fig. 3e, middle left histogram). Likewise, EX527 treatment in combination with RSV 1 nm/d + AMPA did not change spinal cord histology or MN number when compared to AMPA-alone infusion (0.1 ± 0.0 ipsilateral and 9.3 ± 0.9 contralateral versus 0.4 ± 0.3 ipsilateral and 8.7 ± 1.0 contralateral), although this values were significantly different to control values (*p* < 0.05; Fig. 3e, middle left histogram).

QCT ameliorated histological changes in the spinal cord (Fig. 3c), and prevented excitotoxic MN number diminution, especially in the ipsilateral side to the infusion (0.4 ± 0.3 in AMPA versus 6.4 ± 1.3 for QCT 1 nm/d + AMPA, *p* < 0.05; and 4.8 ± 1.1 for QCT 10 nm/d + AMPA, *p* < 0.05; Fig. 3e, middle right histogram). EX527-added infusion to QCT 1 nm/d + AMPA treatment induced a statistically significant reduction in MN number in the ipsilateral side when compared to QCT 1 nm/d + AMPA (1.3 ± 0.7 versus 6.4 ± 1.3, *p* = 0.027, Fig. 3e, middle right histogram).

EX527-alone at 10 nm/d, even after 10 days of infusion, showed a ventral spinal cord histology (Fig. 3e) and MN number similar to control values (Fig. 3e, right histogram). EX527 co-infused with AMPA resulted in spinal cord histology similar to that of AMPA-treated animals (Fig. 3d), and MN number values not different to the AMPA group values (Fig. 3e, right histogram).

### Effects of AMPA, RSV, QCT or EX527 on astrocytes and microglia number

**Fig. 4:**
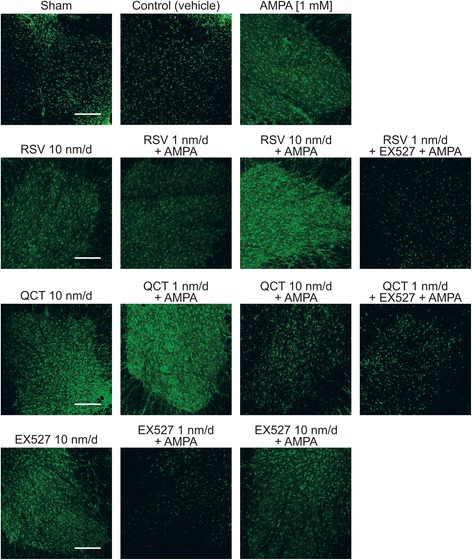
Effect of RSV, QCT and EX527 on spinal astroglial cell number during chronic AMPA-induced excitotoxicity. Representative maximal intensity projections of fluorescent confocal micrographs of GFAP/FITC-immunostained lumbar ventral spinal cord sections of rats contralateral to the side of infusion after 10 days of the indicated treatments. Quantitative analysis is shown in Fig. 5. Scale bar, 200 μm

**Fig. 5:**
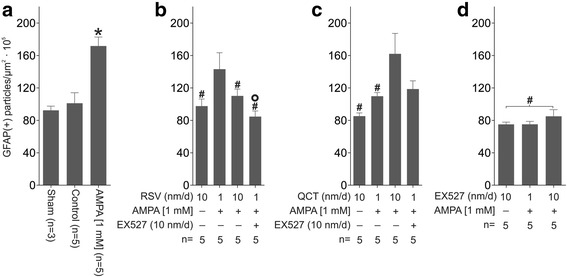
Quantification of GFAP-labeled particles in the ventral horn contralateral to the infusion site. **a** Sham, and control and AMPA infusions. **b** RSV alone or co-infused with AMPA and EX527. **c** QCT alone or co-infused with AMPA and EX527. **d** EX527 alone or co-infused with AMPA. Number of animals is indicated below each column. *p < 0.05 versus control, ^#^ *p* < 0.05 versus AMPA, °*p* < 0.05 versus RSV 1 nm/d + AMPA. One-way ANOVA followed by Tukey’s post hoc test

**Fig. 6:**
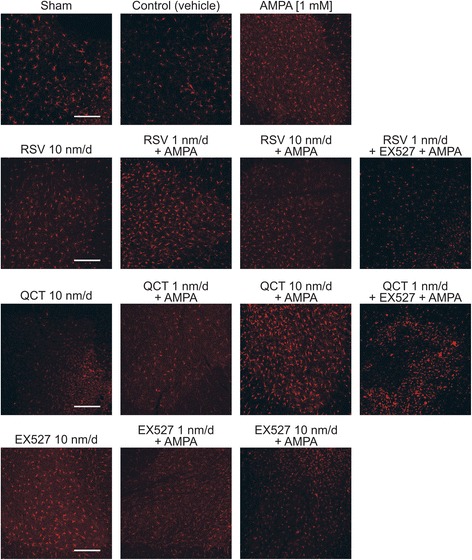
Effect of RSV, QCT and EX527 on spinal microglial cell number during chronic AMPA-induced excitotoxicity. Representative maximal intensity projections of fluorescent confocal micrographs of Iba1/AlexaFluor 546-immunostained lumbar ventral spinal cord sections of rats contralateral to the side of infusion after 10 days of the indicated treatments. Quantitative analysis is shown in Fig. 7. Scale bar, 200 μm

**Fig. 7:**
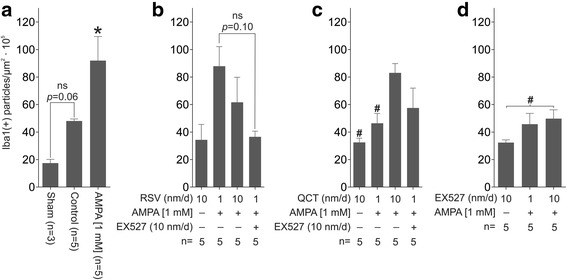
Quantification of Iba1-labeled particles in the ventral horn contralateral to the infusion site. **a** Sham, and control and AMPA infusions. **b** RSV alone or co-infused with AMPA and EX527. **c** QCT alone or co-infused with AMPA and EX527. **d** EX527 alone or co-infused with AMPA. Number of animals is indicated below each column. *p < 0.05 versus control, and ^#^ *p* < 0.05 versus AMPA. One-way ANOVA followed by Tukey’s post hoc test

Glial cell counting in the contralateral side revealed that GFAP(+) particle number does not change in control conditions (vehicle infusion for 10 days) as compared to sham (92.3 ± 5.2 versus 101.1 ± 12.9, *p* = 0.85) (Figs. 4 and 5a). However, we observed a more than 2-fold increase in Iba1(+) particle number, which however did not reach significance (48.0 ± 1.4 versus 17.4 ± 2.7, *p =* 0.06) (Figs. 6 and 7a). We expected this change, since glass capillary insertion into the spinal cord tissue might induce cell death and inflammatory signaling, as has previously been described with stiff materials (such as borosilicate glass) implanted in the rodent brain [28]. AMPA treatment, as expected, was associated with a significant ~2-fold increase, as compared to control group values, in GFAP(+) particle (171.7 ± 11.1 versus 101.1 ± 12.9, Figs. 4 and 5a) and Iba1(+) particle (91.9 ± 17.4 versus 48.0 ± 1.4, Figs. 6 and 7a) number.

RSV-alone infusion (at 10 nm/d) did not change GFAP(+) particle (97.5 ± 8.7 versus 101.1 ± 12.9 in control, *p >* 0.99) or Iba1(+) particle numbers. AMPA-induced GFAP(+) particle number increase was prevented by RSV treatment, only at 10 nm/d (171.7 ± 11.1 versus 110.1 ± 8.3, *p* = 0.03; Figs. 4 and 5b), but Iba1(+) particle number was not altered at neither RSV rate of infusion (87.8 ± 14.2 for 1 nm/d and 61.6 ± 18.2 for 10 nm/d versus 91.9 ± 17.4, *p =* 0.99 and 0.593, respectively; Figs. 6 and 7b). In contrast, adding EX527-infusion to RSV 1 nm/d + AMPA treatment prevented GFAP(+) particle number increase (*p* < 0.05 versus AMPA and *p* < 0.05 versus RSV 1 nm/day + AMPA), although no significant changes in Iba1(+) particle number were attained, even when a clear trend towards a reduction was appreciated.

QCT-alone infusion did not cause changes in glial cell number when compared to control values, either in GFAP(+) particles (85.1 ± 4.0 versus 101.1 ± 12.9, *p* = 0.97) or in Iba1(+) particles (57.4 ± 14.4 versus 48.0 ± 1.4, *p* = 0.87). QCT prevented GFAP(+) and Iba1(+) particle number increases induced by AMPA, although only at the 1 nm/d dose (171.7 ± 11.1 versus 109.8 ± 4.5 for GFAP(+) particle number, *p* = 0.018, Figs. 4 and 5c; and 46.3 ± 7.1 versus 91.9 ± 17.4 for Iba1(+) particle number, *p =* 0.049, Figs. 6 and 7c), while the larger dose did not have any effect. Adding EX527-infusion to QCT 1 nm/d + AMPA treatment did not significantly reduce the GFAP(+) particle (118.6 ± 10.1 versus 109.8 ± 4.5 in QCT 1 nm/day + AMPA, *p* = 0.99) or Iba1(+) particle (*p* = 0.96 versus QCT 1 nm/day + AMPA) number, although a clear trend is evident.

EX527-alone treatment resulted in glial number similar to control values (75.0 ± 2.8 versus 101.1 ± 12.9, *p* = 0.28 for GFAP(+) particles, Figs. 4 and 5d; 32.3 ± 2.0 versus 48.0 ± 1.4, *p* = 0.66 for Iba1(+) particles, Figs. 6 and 7d). GFAP(+) and Iba1(+) particle numbers resulted markedly reduced by EX527 treatment in AMPA-induced excitotoxic conditions, without a dose-related effect, reaching values similar to control conditions (*p* < 0.01 versus AMPA for GFAP(+) particle quantification, and *p* < 0.05 versus AMPA for Iba1(+) particle quantification).

## Discussion

In this work we investigated the effects of the polyphenols RSV and QCT in our model of excitotoxic MN death and, since polyphenols’ efficacy has been attributed to SIRT1 activation [29], we also addressed the involvement of this sirtuin activity to the effects observed.

SIRT1 was located in the nuclei and cytoplasm of neurons, including those of MNs, of the lumbar spinal cord of intact rats. These results are similar to those recently reported in the spinal MNs of transgenic hSOD1^G93A^ mouse [30], whereas data from another group showed that SIRT1 is widely expressed in the brain and spinal cord of healthy humans and rodents, but restricted to the nuclei of neurons [31]. In post-mortem spinal cord tissue of ALS patients SIRT1 mRNA is increased [32], whereas in the hSOD1^G93A^ mouse a study reported increased levels of SIRT in the spinal cord [33], and according to another report this level decreases with disease progression [34]. We did not observe SIRT1 immunolabeling in astrocytes and microglial cells, which differs from the finding that SIRT1 is expressed in cultured mouse astrocytes [35], so this differences might be due to the different species and experimental conditions (in vivo and in vitro). In agreement, an exhaustive study of the expression pattern of sirtuins in the rat nervous tissue found that SIRT1 is present at low concentrations in whole tissue homogenates of the spinal cord, while in vitro is more abundant in neurons than in astrocytes [36].

RSV, classically described as a STAC that preferentially targets SIRT1 [37], did not exert significant protection against AMPA-induced paralysis or MN loss, although it prevented the AMPA-induced increase in the number of astrocytes, while it had no effect on microglial cell number. This was an unexpected result, given the numerous works supporting its potential as a protective agent in models of excitotoxic neuronal death. For example, RSV prevented glutamate-induced neuronal death in brain slices cultures [38], as well as neuronal loss in a model of kainate-induced brain injury in vivo [39]. However, few studies have explored the role of RSV in experimental models of MN loss. RSV was shown to reduce spontaneous degeneration of primary cortical neurons transfected with hSOD1^G93A^ [40], and to upregulate SIRT1 expression in a culture of hSOD1^G93A^-transfected MN-like cells [41]. This was corroborated in vivo in two studies*,* where RSV extended the survival of the transgenic hSOD1^G93A^ mice [30, 42], a result attributed to an upregulation of SIRT1 activity and to a prevention in spinal microgliosis and astrogliosis, as well as to an improvement of mitochondrial respiratory function. Although in the present work we did not address mitochondrial function, previous research from our group support the notion that AMPA-induced excitotoxicity involves energy disruption. Indeed, energy substrates administration prevents the alterations of mitochondrial respiratory complexes induced by AMPA in the spinal cord, and this is associated with MN survival and improved motor behavior performance [14, 15]. These findings reinforce the idea that AMPA-mediated chronic excitotoxicity and expression of hSOD1^G93A^ share common mechanisms that prompts MN degeneration.

Regardless of these similarities, RSV was not neuroprotective under our experimental conditions. In this respect, SIRT1 activation by RSV was not confirmed in the studies mentioned previously [30, 40–42], which might imply that RSV effects could be unrelated to SIRT1 expression or activation. In fact, results from recent work suggest that RSV might not be a STAC [43], and that other targets merit consideration, such as AMP-activated protein kinase (AMPK) [44]. Further, it has been suggested that the neuroprotective actions of RSV are not related to SIRT1 activation [45]. We propose that the activation of other molecular pathways by RSV, such as AMPK, might be deleterious for MN survival under AMPA-induced excitotoxic conditions in vivo*,* as has been shown in the hSOD1^G93A^ transgenic mice [46]. In agreement, it was recently reported that RSV did not protect hippocampal neurons in rat pups treated with kainic acid [47].

QCT exerted a remarkable effect preventing AMPA-induced paralysis, as evidenced by the performance in the rotarod test, as well as a moderate reduction in MN loss. These results were independent of the dose used, and were significant even with the lowest dose of 1 nanomole/day. QCT also diminished the astrogliosis and microgliosis induced by AMPA, but only at the lower dose. Previous studies have demonstrated that QCT is neuroprotective under excitotoxic conditions, albeit these results have been ascribed to the antioxidant capacity of QCT. For example, QCT protected against neuronal death and mitochondrial dysfunction caused by N-methyl-D-aspartate and adenosine diphosphate plus iron in vitro [48]. Also, in a model of cerebral ischemia-reperfusion injury in vivo, QCT diminished infarct volume, improved behavioral deficits and reduced the levels of nitrite and malonyl dialdehyde, both markers of oxidant stress [22]. Whether QCT is a direct scavenger of reactive oxidant species or activates molecular pathways related to oxidant defenses was not addressed in these studies. We consider that the results observed in the present work are independent of QCT antioxidant activity, because we have shown previously that under similar experimental conditions reactive nitrogen species are nearly absent in spinal cord tissue [49] and, more importantly, that classical antioxidant molecules, such as glutathione and ascorbate, do not confer protection against neither acute [15] or chronic [14] excitotoxic damage to spinal cord MNs.

Data on the effects of QCT in the MND setting are very scarce. QCT increased survival of motor neuron 2 gene mRNA in cultured fibroblasts from patients affected by SMA [50], and increased survival of cultured lymphocytes obtained from familial ALS patients caused by the mutated SOD1 [51]. To the best of our knowledge, no studies investigating QCT effects in MND models have been carried out. However, the survival of embryonic spinal MNs from rats was increased by QCT [52].

Interestingly, we found that QCT beneficial effects were partially suppressed by the specific inhibitor of SIRT1, EX527, and that SIRT1 is located in neuronal cells in the spinal cord, specifically in their nucleus and cytoplasm, similarly to previous findings [30]. These results lead us to propose that QCT activates SIRT1, and that this is probably the mechanism of its protective action, rather than its antioxidant activity, as discussed above. In agreement, QCT-mediated survival in cultured neurons in a model of herpes simplex virus 1 infection was ascribed to the increase in SIRT1/AMPK axis activation [53]. In a model of hypobaric-hypoxic brain injury in vivo, QCT prevented cognitive deterioration through SIRT1 upregulation and consequent activation of peroxisome proliferator-activated receptor gamma coactivator 1-alpha (PGC1-α), one of the master regulators of mitochondrial biogenesis [23]. Further, in an in vitro model of dopaminergic neuronal death induced by 6-hydroxydopamine and MitoPark, QCT prevented neuronal death through the activation of the protein kinase D/cAMP response element binding protein/PGC1-α axis, which led to the augmentation of mitochondrial biogenesis and function [54]. These studies suggest that QCT may exert its beneficial effects ultimately improving neuronal energetic state irrespective of its molecular targets.

How does SIRT1 activation protect endangered neurons? Intense research has evidenced that SIRT1 is involved in a vast amount of molecular pathways, most of them intertwined in a complex signaling network that links energetic and metabolic stress to changes in gene expression through epigenetic modifications (histone deacetylation) and modulation of the transcriptional machinery (for a review on the subject see [55]). Which signaling pathway is activated by SIRT1 depends on the experimental model and conditions. Given the relative lack of specificity of the polyphenols, genetic strategies are most suitable to specifically study SIRT1. For example, in a study of double transgenic mice that overexpress SIRT1 in the central nervous system and also harbor the hSOD1^G93A^ mutation, an increased survival was observed, partly due to activation of the heat shock factor 1/inducible heat shock protein 70 chaperone system [56].

Interestingly, the chronic infusion of the SIRT1 inhibitor EX527 alone did not affect motor behavior or induced MN loss. Although SIRT1 expression in the hippocampus seems essential for normal cognitive function and synaptic plasticity in mice [57], no studies have investigated the role of SIRT1 in spinal cord physiology. We hypothesized that EX527 infusion would enhance excitotoxic MN loss and paralysis, since SIRT1 activity would be impaired. Surprisingly, no additional motor behavioral deficits or MN degeneration were observed. Robust experimental evidence in vitro has pointed out that excitotoxicity unequivocally leads to energetic stress, commensurate with reducing amounts of NAD(P)^+^ [58, 59]. Since NAD^+^ is SIRT1’s required substrate for its catalytic activity [60], NAD^+^ depletion in excitotoxic conditions would result in SIRT1 activity downregulation, which will affect the signaling pathways necessary for survival of the stressed neurons, as has been recently shown [61]. In contrast, SIRT1 activity could also have deleterious effects: consumption of NAD^+^ in an already energetically compromised neuron may trigger cell death pathways activation, for example through enhanced poly(ADP)ribose polymerase activity [62]. In fact, recent studies have pointed out that SIRT1 inhibition (with nicotinamide) or knock-down may be beneficial for neuronal survival during excitotoxicity [63, 64] or oxidative stress [65]. Therefore, we propose that the lack of enhancement of MN death by EX527 might by due to an increased availability of NAD^+^ for other prosurvival pathways, such as energy restoration, as has been suggested in an in vivo model of prion-related neurodegeneration [66].

Besides paralysis and MN loss, we quantitatively confirmed that AMPA infusion induces an increase in astroglial and microglial cells in the spinal cord. This finding was previously reported by our group, but only qualitatively [12]. Numerous studies have already established the crucial role of glial cells in MN degeneration [67]. Our present findings suggest that the abundance of astroglial and microglial cells is not related to neuronal protection, since QCT protection was not accompanied by a reduction in glial cell number. This implies that the molecular pathways modulated by RSV and QCT operate independently on MNs and glial cells, which argues against non-autonomous cell death mechanisms [68].

## Conclusions

In summary, chronic AMPA-mediated excitotoxicity in the lumbar spinal cord in vivo induces MN death, manifested as hindlimb paralysis, as well as astrogliosis and microgliosis. The SIRT1 activator QCT prevents excitotoxic MN death, paralysis and gliosis, in part through a SIRT1-dependent mechanism, because the specific inhibitor EX527 partially suppressed QCT-exerted neuroprotective effects. RSV and EX527 had no effect on the AMPA-mediated excitotoxic spinal neurodegeneration. These results contribute to establish polyphenols as promising therapeutic targets in neurodegeneration, and offer insights into their possible mechanisms of action.

## Additional file

Additional file 1: Figure S1. Procedure for the chronic infusion of drugs through osmotic minipumps into the L3-L4 segment of the spinal cord and effect of the drugs used. **(A)** Schematic representation of the site of infusion in the rat spinal cord (red dot), viewed from the animal’s dorsum; the innervated hindlimb muscles by the respective spinal segments is indicated. **(B)** Representative low-magnification photomicrographs of Nissl stained lumbar spinal cord slices after 10 days of continuous infusion of the indicated treatments. Black arrows indicate the site of the cannula insertion. Note the effects of treatments (nm/d indicates nanomoles/day) on the MNs in the ventral horns, which are shown at higher magnification in Fig. 3. Scale bar, 500 μm. **(C)** Representative high magnification photomicrographs of the healthy MNs of the contralateral side of an AMPA-treated rat (left) and of the degenerating MNs (right, white arrows) of the corresponding infused side (marked by squares in the low magnification micrograph of the AMPA-treated spinal cord). Scale bar, 100 μm. (JPEG 2065 kb)

## Abbreviations

ALS: Amyotrophic lateral sclerosis
AMPA: α-Amino-3-hydroxy-5-methyl-4-isoxazolepropionic acid
AMPK: Adenosine mononucleotide phosphate-activated protein kinase
GFAP: Glial fibrillary acidic protein
hSOD1^G93A^: Human mutated superoxide dismutase, with substituted glycine to alanine in position 93
Iba1: Ionized calcium-binding adapter molecule 1
MAP2: Microtubule associated protein 2
MN: Motor neuron
MNDs: Motor neuron disorders
NAD, NAD(P): Nicotine adenine dinucleotide (phosphate)
QCT: Quercetin
RSV: Resveratrol
SIRTs: Sirtuins
SMA: Spinal muscular atrophy
STAC: Sirtuin activating compound
