# Supplementary figures and images for: Quercetin prevents spinal motor neuron degeneration induced by chronic excitotoxic stimulus by a sirtuin 1-dependent mechanism
Source: Transl Neurodegener. 2017 Nov 21;6:31. doi: 10.1186/s40035-017-0102-8 (PMC5697078; doi:10.1186/s40035-017-0102-8)

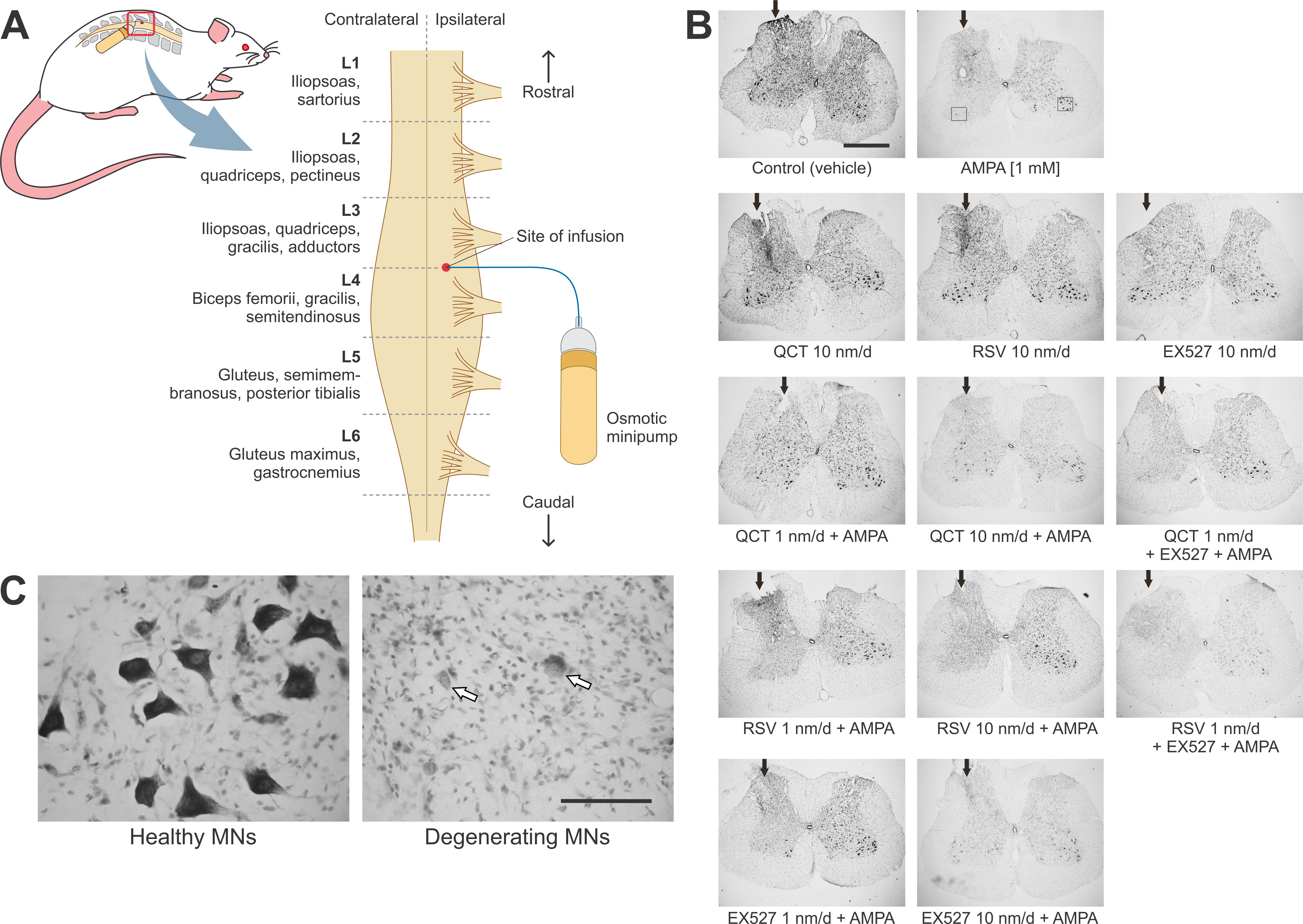

Supplement: Additional file 1: Figure S1. — Procedure for the chronic infusion of drugs through osmotic minipumps into the L3-L4 segment of the spinal cord and effect of the drugs used. (A) Schematic representation of the site of infusion in the rat spinal cord (red dot), viewed from the animal’s dorsum; the innervated hindlimb muscles by the respective spinal segments is indicated. (B) Representative low-magnification photomicrographs of Nissl stained lumbar spinal cord slices after 10 days of continuous infusion of the indicated treatments. Black arrows indicate the site of the cannula insertion. Note the effects of treatments (nm/d indicates nanomoles/day) on the MNs in the ventral horns, which are shown at higher magnification in Fig. 3. Scale bar, 500 μm. (C) Representative high magnification photomicrographs of the healthy MNs of the contralateral side of an AMPA-treated rat (left) and of the degenerating MNs (right, white arrows) of the corresponding infused side (marked by squares in the low magnification micrograph of the AMPA-treated spinal cord). Scale bar, 100 μm. (JPEG 2065 kb) [file 40035_2017_102_MOESM1_ESM.jpg]
